# A case report of native vertebral osteomyelitis caused by *Cutibacterium modestum*

**DOI:** 10.1186/s12879-022-07341-2

**Published:** 2022-04-11

**Authors:** Taiji Koyama, Goh Ohji, Masako Nishida, Sho Nishimura, Iku Shirasugi, Kenichiro Ohnuma, Mari Kusuki, Kentaro Iwata

**Affiliations:** 1 Department of Medical Oncology and Hematology, Kobe University Hospital, 7-5-2, Kusunoki-cho, Chuo-ku, Kobe, Hyogo Japan; 2 Department of Clinical Laboratory, Kobe University Hospital, 7-5-2, Kusunoki-cho, Chuo-ku, Kobe, Hyogo Japan; 3 Department of Infectious Diseases, Kobe University Hospital, 7-5-2, Kusunoki-cho, Chuo-ku, Kobe, Hyogo Japan; 4 Department of Rheumatology, Kobe University Hospital, 7-5-2, Kusunoki-cho, Chuo-ku, Kobe, Hyogo Japan

**Keywords:** *Cutibacterium*, Vertebral osteomyelitis, Biochemical analysis, MALDI-TOF

## Abstract

**Background:**

*Cutibacterium modestum* was named in 2020. *C. modestum* was previously called *Propionibacterium humerusii*. Several implant-associated infections caused by Cutibacterium species have been previously reported, but native vertebral osteomyelitis due to these bacteria has rarely been reported.

**Case presentation:**

A 72-year-old man, who had previously received several nerve block injections for low back pain, was referred to our hospital for deterioration in back pain in the last 1 month. MRI findings were suggestive of L5-S1 vertebral osteomyelitis. Blood cultures and bone biopsy culture revealed the presence of Gram-positive bacilli. The isolate was identified as *C. modestum* by 16SrRNA gene sequencing. A diagnosis of vertebral osteomyelitis caused by *C. modestum* was made. Minocycline followed by oral amoxicillin was administered for 3 months. His symptom improved and did not recur after treatment completion.

**Conclusion:**

A case of vertebral osteomyelitis caused by *C. modestum* was encountered. Although *C. modestum* is very similar to *C. acnes*, it could be accurately identified by 16SrRNA gene sequencing. This case represents the first documented *C. modestum* infection in humans.

## Background

The genus *Cutibacterium* was previously called *Propionibacterium*. *Cutibacterium* is a Gram-positive anaerobic bacterium that is a significant component of the human skin microbiota. A new species of Propionibacterium was reported in 2011 and named *Propionibacterium humerusii* [1]. Dekio and colleagues proposed renaming this bacterium *Cutibacterium modestum*. Here, we report the first documented *C. modestum* infection not associated with implant or direct medical procedure.

## Case presentation

**Fig. 1:**
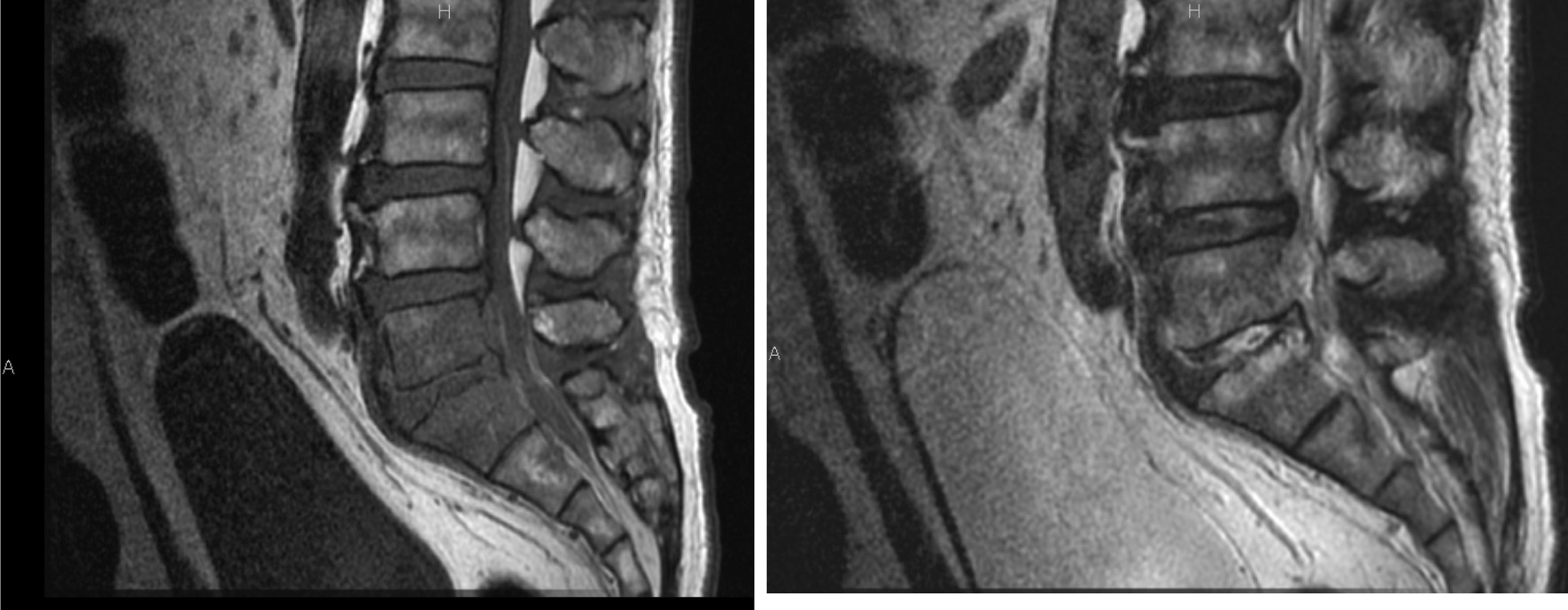
T1-weighted MRI (Left) and T2-weighted MRI (Right) images of suspected osteomyelitis at L5 and S1. T1-weighted MRI (Left) and T2-weighted MRI (Right) showed that intensity of L5 and S1 vertebrae were altered and vertebral osteomyelitis was suspected

A 72-year-old Japanese man was referred to our hospital for treatment of vertebral osteomyelitis. He had been followed by his primary care physician for lumber spinal canal stenosis and type 2 diabetes mellitus. He had received several nerve block injections for his low back pain. One month prior to admission the patient’s low back pain worsened when he visited his family physician. Magnetic resonance imaging (MRI) was performed, and findings were suggestive of L5-S1 vertebral osteomyelitis (Fig. 1).

**Fig. 2:**
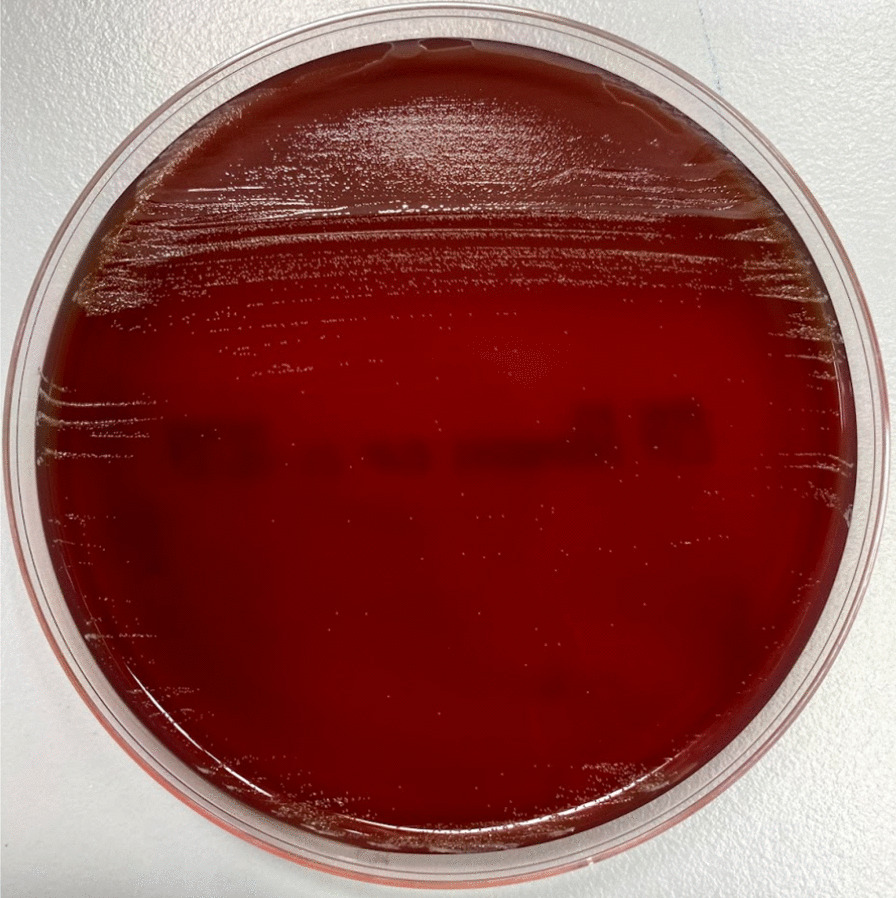
Culture colonies observed on Brucella HK agar after 48 h of incubation. After 48 h of incubation, small colonies were observed on Brucella HK agar from blood culture

On the day of admission, the patient’s body temperature was 37.5 °C and all of his vital signs were within their normal reference ranges. The patient’s back pain worsened by the straight leg raise test and he had no neurologic deficits. His skin appearance around the L5-S1 vertebrae was normal. His lab values included a white blood cell count of 9.6 × 10^9^ cells/L, hemoglobin of 150 g/L, platelet count of 31.7 × 10^9^ cells/L, and C-reactive protein level of 139 mg/L. Computed tomography confirmed that there was no other site of infection other than at the lumbosacral junction, and transthoracic echocardiography did not find any evidence of endocarditis. Two sets of blood cultures were taken from different site on admission day. Each set contains an aerobic bottle and an anaerobic bottle. One anaerobic bottle turned out to be positive for Gram-positive bacilli on hospital day 7. Since we thought this Gram-positive bacilli might be a contamination, we treated him with only non-steroidal anti-inflammatory drugs on this time. Hospitalist also consulted to physical medicine and rehabilitation team. To confirm the causative organism of vertebral osteomyelitis percutaneous CT-guided needle biopsy on hospital day 8. Growth of Gram-positive bacilli was observed on the hemin and vitamin K1 (HK) semi-solid medium (Kyokuto Pharmaceutical Industrial Co., Ltd, Japan) of the biopsy culture. And Gram-positive bacilli from blood culture was also grown on the same medium. Non-hemolytic small colonies were observed on the Brucella HK agar from blood culture after 48 h of incubation under anaerobic conditions (Fig. 2). The isolated organism was identified using matrix-assisted laser desorption ionization-time of flight mass spectrometry (MALDI-TOF MS) with MALDI Biotyper Compass Version 4.1.70 software (Bruker Daltonik GmbH, Bremen, Germany). MALDI-TOF MS were difficult to identify the specimen, and the one of possibility bacterium was *C. acnes*. However, unlike *C. acnes*, this organism had a negative reaction to N-acetyl-β-glucosaminidase, proline arylamidase, glycine arylamidase (Table 1).

**Table 1:** Comparison of the biochemical findings of the novel bacteria compared with *Cutibacterium acnes*

Enzyme	Isolate		C. acnes (positive, %)	
	RapidID 32A (bioMérieux)	ANA II (Innovative Diagnostic Systems)	RapidID 32A (bioMérieux)	ANA II (Innovative Diagnostic Systems)
Urease	−	−	0	0
Arginine dihydrolase	Weak	N/A	62	N/A
α-Galactosidase	−	−	0	0
β-Galactosidase	−	−	69	5
β-Galactosidase-6-phosphate	−	N/A	0	N/A
α-Glucosidase	−	−	23	52
β-Glucosidase	−	−	0	0
α-Arabinosidase	−	−	0	0
β-Glucuronidase	−	N/A	0	N/A
N-Acetyl-β-glucosaminidase	Weak	Weak	90	88
Glutamic acid decarboxylase	−	N/A	0	N/A
α-Fucosidase	−	−	0	0
Arginine arylamidase	+	+	88	98
Proline arylamidase	−	−	100	98
Leucylglycine arylamidase	−	−	54	96
Phenylalanine arylamidase	−	−	8	48
Leucine arylamidase	−	N/A	69	N/A
Pyroglutamic acid arylamidase	−	−	1	71
Tyrosine arylamidase	−	N/A	8	N/A
Alanine arylamidase	−	N/A	85	N/A
Glycine arylamidase	−	−	91	98
Histidine arylamidase	−	N/A	8	N/A
Glutamyl glutamic acid arylamidase	−	N/A	0	N/A
Serine arylamidase	−	N/A	69	N/A
Mannose	+	N/A	46	N/A
Raffinose	−	N/A	0	N/A
Nitrate reduction	Weak	N/A	85	N/A
Indole	Weak	Weak	62	85

*N/A* not applicable

To further characterize and identify the isolated organism, 16SrRNA gene sequencing using universal primers was performed as previously reported [2]. A Basic Local Alignment Search Tool (BLAST) search (www.ncbi.nlm.nih.gov/BLAST) for the 16S rRNA gene sequencing was performed using the taxonomy browser of the National Center for Biotechnology Information. The sequence result showed 100% similarity (1366/1366 bp) with a strain of *Propionibacterium humerusii* P08 (accession No. AFAM00000000.1); therefore, the isolate was identified as *P. humerusii* (*C. modestum*).

Prior to the precise identification of this organism with BLAST, the minimum inhibitory concentration of antibiotics was assessed with a broth microdilution method using Brucella broth (Eiken Co.) under anaerobic conditions (Table 2). According to these results we started intravenous minocycline therapy on hospital day 31. After correctly identifying the isolate, in reference to the European Committee on Antimicrobial Susceptibility Testing (EUCAST) breakpoints for *C. acnes* and Gram-positive anaerobes, we switched the antibiotics to oral amoxicillin. Hospitalist consulted to physical medicine and rehabilitation team. Patient was transferred to another hospital and continue physical therapy and rehabilitation. We continued amoxicillin for 3 months. The patient’s symptoms improved and did not recur 2 years after treatment completion.

**Table 2:** The minimum inhibitory concentration (MIC) of antibiotics of the novel bacterium

Antibiotics	MIC
Ampicillin	≦ 0.25
Ceftriaxone	≦ 8
Meropenem	≦ 8
Ampicillin/sulbactam	≦ 4/2
Piperacillin/tazobactam	≦ 16/4
Clindamycin	2
Minocycline	≦ 1
Moxifloxacin	≦ 2

## Discussion and conclusions

*Cutibacterium modestum* was previously described as “*Propionibacterium humerusii*.” The DNA sequence of *C. modestum* is 89% similar to that of *C. acnes* [1]. Previous studies have reported that this bacterium can be detected in human skin [3, 4]. This organism was formally termed as “*Cutibacterium modestum*” in 2020 [5].

MALDI-TOF MS is widely used for bacterial identification and allows for the relatively easy and quick identification of microorganisms, including *C. acnes*. However, the predominant peaks on mass spectrometry of *C. modestum* are different compared with those of *C. acnes* and its subspecies [6]. MALDI-TOF MS originally suggested that our isolate was *C. acnes*. However, the log score 1.62 of this species was not adequate to accurately identify the bacteria on either the species or genus level. In addition, the biochemical qualities of this isolate, in particular glycine arylamidase and indole levels, were not consisting with those of *C. acne*s and other Cutibacterim species [7]. We therefore performed 16SrRNA sequencing of the isolate. Biochemical analysis was very important for distinguishing *C. modestum* from other Cutibacterium species.

Since the description of *Propionibacterium humerusii* in 2011 and its new name *C. modestum*, no literature has reported a clinical *C. modestum* infection in humans. We were able to successfully treat this patient using antibiotics alone in accordance with the EUCAST breakpoint for *C. acnes* and Gram-positive anaerobes [8]. However, whether our choice of antibiotic was appropriate is uncertain. Accumulation of clinical experience of human infection caused by *C. modestum* is required to answer this question.

Recently, an implant-associated *C. modestum* infection was reported [9]. Our case patient was diagnosed as native vertebral osteomyelitis. Implant-associated *C. acnes* infections have been previously reported [10, 11], as well as Cutibacterium species-related native vertebral osteomyelitis.

Growth of Cutibacterium species depends on the bacterial inoculum size. It took 7 days for blood culture growth in our case. This suggests low inoculum of bacteremia in this case. When Cutibacterium species is considered as causative pathogen, prolonged blood culture incubation might be feasible.

In conclusion, we reported the native vertebral osteomyelitis due to *C. modestum*. *C. modestum* is very similar to *C. acnes*, and may be misidentified as *C. acnes*. The biochemical characteristics and inadequate results of MALDI-TOF were very important for distinguishing this bacterium from other Cutibacterium species. Further microbiological and clinical investigations are required to better describe the management of *C. modestum* infections.

## Data Availability

The sequence determined by the 16SrRNA gene analysis of the *C. modestum* strain in our case is available in the International Nucleotide Sequence Database through the DNA Databank of Japan under the accession number LC414574. The datasets used and/or analyzed during the current study are available from the corresponding author on reasonable request. The sequencing data is available in NCBI GenBank, https://www.ncbi.nlm.nih.gov/nuccore/LC414574.1.

## Abbreviations

*C. modestum*: *Cutibacterium modestum*
*C. acnes*: *Cutibacterium acnes*
MRI: Magnetic resonance imaging
HK: Hemin and vitamin K1
MALDI-TOF MS: Matrix-assisted laser desorption ionization-time of flight mass spectrometry
BLAST: Basic Local Alignment Search Tool
*P. humerusii*: *Propionibacterium humerusii*
EUCAST: European Committee on Antimicrobial Susceptibility Testing
